# Real-time robot topological localization and mapping with limited visual sampling in simulated buried pipe networks

**DOI:** 10.3389/frobt.2023.1202568

**Published:** 2023-11-23

**Authors:** Xiangyu S. Li, T. L. Nguyen, Anthony G. Cohn, Mehmet Dogar, Netta Cohen

**Affiliations:** ^1^ School of Computing, University of Leeds, Leeds, United Kingdom; ^2^ Alan Turing Institute, London, United Kingdom; ^3^ Tongji University, Shanghai, China; ^4^ Shangdong University, Jilin, China

**Keywords:** pipe networks, topological mapping, localization, autonomous control, robot simulation

## Abstract

**Introduction:** Our work introduces a real-time robotic localization and mapping system for buried pipe networks.

**Methods:** The system integrates *non-vision-based* exploration and navigation with an *active-vision-based* localization and topological mapping algorithm. This algorithm is selectively activated at topologically key locations, such as junctions. *Non-vision-based* sensors are employed to detect junctions, minimizing the use of visual data and limiting the number of images taken within junctions.

**Results:** The primary aim is to provide an accurate and efficient mapping of the pipe network while ensuring real-time performance and reduced computational requirements.

**Discussion:** Simulation results featuring robots with fully autonomous control in a virtual pipe network environment are presented. These simulations effectively demonstrate the feasibility of our approach in principle, offering a practical solution for mapping and localization in buried pipes.

## 1 Introduction

The development of indoor robot localization technology has gained substantial interest from researchers, industry, and a large user base (Jensfelt, 2001). Pipeline localization is also witnessing increased attention, driven by the need for more efficient and cost-effective maintenance and repair of critical infrastructure such as oil and gas pipelines, water and sewage systems, and electrical transmission lines (Mounce et al., 2021). However, unlike most indoor settings, there remain significant challenges in miniaturizing and making pipeline robots autonomous. Such autonomous robots must have accurate and dependable sensors for data collection and decision-making, as well as efficient and compact power sources and actuators. Inspection, localization and navigation algorithms must operate in real-time, while also being computationally efficient to reduce power requirements and minimize hardware needs. Existing infrastructure maps may not accurately represent the current state of pipeline networks due to changes that occur over time. As a result, relying solely on traditional maps can lead to outdated information, potentially hindering maintenance and repair operations. To overcome this challenge, autonomous mapping techniques offer a solution by providing real-time updates, ensuring the most accurate and up-to-date information. However, accurate localization and navigation are crucial for robots to efficiently maneuver through the pipeline network and carry out tasks at specific locations. Traditional maps, which primarily focus on geometric features, may not provide sufficient information for autonomous robot navigation and decision-making. This limitation necessitates the use of topological maps that capture not only the geometric features but also the connectivity and relationships between pipeline components, such as junctions and manholes. By leveraging topological maps, robots can plan optimal paths, navigate complex networks, and perform advanced computations, such as semantic reasoning and planning. Therefore, to address these challenges and improve the efficiency and effectiveness of pipeline inspection and maintenance, we propose a topological mapping algorithm. Topological representations are compact as well as straightforward and intuitive (Kortenkamp and Weymouth, 1994), providing a clear understanding and interpretation of the environment. Indoor robots utilize mapping and localization to determine their position within an environment, even when direct or relative location sensing is unavailable. A commonly used technique is Simultaneous Localization and Mapping (SLAM), which employs geometric representations to construct maps of unknown environments while simultaneously determining the robot’s location on the map (Mur-Artal et al., 2015; Rugg-Gunn and Aitken, 2022).

Compared with a map of geometric features, a topological map is more efficient in terms of storage and computation, and insensitive to error accumulation. Topological maps provide an abstraction of the physical environment and a more sophisticated spatial representation. These abstractions are well suited to built environments, both indoor and underground, allowing robotic systems to perform advanced computations such as semantic reasoning and planning. Furthermore, as suggested by Chrastil and Warren (2013), topological maps align with human spatial intuition, facilitating more natural human-computer interactions. Our work presents an algorithm for topological localization and mapping in buried pipe networks, enabling autonomous navigation for lightweight robots over extended periods.

We present a hybrid approach integrating *vision-* and *non-vision-based* strategies in a single fully autonomous framework. In particular, we use *non-vision-based* exploration and navigation while our localization and mapping rely on an active-vision algorithm, which is only activated at topologically key (landmark) locations. Nguyen et al. (2022) describe an autonomous robot for pipeline inspection which they use to demonstrate exhaustive *non-vision-based* exploration of a physical pipe network. The small size of the robot (70 g, navigating through 150 mm pipes) imposes severe limitations on sensors, motors and battery power. Instead of using a camera, the robot uses distance sensors to navigate through the pipes and to execute turns and maneuvers at junctions and dead ends. Here, we propose to add localization and mapping capabilities to this platform. We implement this robot in a simulated pipe network to develop and test our active vision, localization and mapping algorithm. In our work, we focus primarily on junctions, as these are sufficient for the topological mapping of the network. Once the robot arrives at a junction, it uses its camera to collect an image dataset of the location. The robot localizes by comparing the images from its current location with the data in its database. This localization step includes orientation matching, i.e., calculating the robot’s rotation relative to the orientation in the database. An active vision step is included to increase the accuracy and robustness of the localization. Finally, the robot either identifies its location based on a good match with an existing location in the database or defines its location as novel, adding the location information and the associated image set to the database and the topological map.

This paper is organized as follows. The remainder of this section discusses related work. In addition to the creation of topological maps such as mentioned above, we have also developed control algorithms for the fully autonomous navigation of the robot in our experiments, so that the robot can generate topological maps in real-time as it explores the pipeline. The methods are described in Section 2, including a brief overview of the autonomous control of the simulation robot presented by Nguyen et al. (2022) (Section 2.1). Although the physical robot was successful in exploring the pipe network, this study relies on a motorized camera that has yet to be implemented. Hence, the experiments with localization and topological mapping are conducted in a simulated environment. The results are presented in Section 3, including simulations of the robot recognizing junctions in real-time and constructing a topological map as it moves through the pipeline (Section 3.6). Finally, the paper concludes with a discussion in Section 4.

### 1.1 Related work

Localization in robotics involves both relative and absolute positioning methods. Absolute positioning relies on directly obtaining coordinate information through methods like GPS or satellite positioning (El-Rabbany., 2002). However, such methods are not available in the underground pipe networks and therefore are not useable. While relative positioning, also known as dead reckoning, estimates the current position relative to the initial point using sensors such as wheel encoders (Aqel et al., 2016). However, odometry, a common method for dead reckoning in wheeled robots, has limitations such as tire slippage, meandering trajectories, and intrinsic noise (Wang et al., 2015). On the other hand, lidar and camera vision positioning technologies offer better stability and accuracy, but each has its advantages and disadvantages (Nistér et al., 2004; Wang et al., 2017).

In the context of pipeline localization, various approaches have been proposed. Hertzberg and Kirchner (1996) introduced landmark features in pipelines, although the reliability of landmark classification may be questionable. Vision-based localization has been implemented in pipelines by leveraging topological features such as elbows and branches (Lee et al., 2009). These approaches utilize image analysis to extract information about landmarks and update the robot’s records for localization purposes. Some studies have focused on utilizing onboard sensors, such as IMU, gyro, and leak sensors, to localize robots in water pipes (Wu et al., 2019). Other works combine visual odometry and pipeline topological mapping for localization in sewage systems (Alejo et al., 2019).

Localization techniques have been employed in diverse robotics environments. Lopes et al. (2020) developed a reconfigurable robotic miner prototype that utilizes various sensing techniques, including tactile, conductivity, and inertial measurements, for localization and mapping during mining operations. They employ advanced probabilistic sensor fusion techniques for localization in the absence of a globally fixed coordinate frame. Topological mapping and localization have also been extensively studied in urban environments (Kortenkamp and Weymouth, 1994; Blaer and Allen, 2002).

Kalman filter-based methods have been widely utilized for sensor data fusion in pipeline navigation. Siqueira et al. (2016) proposed a sensor data fusion technique using the Kalman filter for pipe navigation. Liu and Krys (2012) explored the use of a laser range finder for pipe inspection, while Maneewarn and Thung-od (2015) presented the ICP-EKF localization method for boiler inspection robots. These studies collectively enhance our understanding of sensor data fusion, laser range finder utilization, and localization algorithms, providing valuable insights for the development of innovative pipe inspection robotics solutions.

SLAM techniques have been successfully applied in pipeline environments. PipeSLAM, introduced by Ma et al. (2017), addresses the challenges of feature-sparse water pipes by utilizing the Rao-Blackwellized particle filter to integrate odometry and visual information. Additionally, Krys and Najjaran (2007) developed a VSLAM system for pipe inspection robots, demonstrating the effectiveness of visual-based methods in pipeline localization. By combining visual information and SLAM techniques, the robot can simultaneously map the pipe environment and estimate its position.

Node localization in robotic sensor networks and the development of smart wireless robotic systems have been significant areas of research in pipeline inspection. Wu et al. (2015) focused on node localization in robotic sensor networks for pipeline inspection. They proposed techniques and algorithms to accurately determine the location of nodes within the network, enabling effective monitoring and control of robotic systems in pipeline environments. Kazeminasab and Banks (2022a) introduced a size-adaptable in-pipe wireless robotic system. This system incorporated a two-phase motion control algorithm to navigate and inspect water distribution systems. By integrating wireless communication capabilities, smart sensors, and adaptable motion control algorithms, the SmartCrawler system demonstrated advancements in the field of pipeline inspection, enhancing the efficiency and effectiveness of water distribution system monitoring and maintenance.

Acoustic echo localization has emerged as a promising technique for pipe inspection robots in recent years. Worley et al. (2020) focused on leveraging acoustic echoes to estimate the location and geometry of pipes accurately. By analyzing the time-delay and frequency characteristics of reflected acoustic signals, the proposed method provided valuable information for localization and mapping tasks in pipe inspection. This work offering a non-intrusive and efficient approach to gather essential information about pipe conditions and geometries.

Kazeminasab et al. (2020) proposed various techniques that integrated sensor data, such as odometry and inertial measurements, with map information to achieve reliable robot localization within the intricate pipe network. Additionally, magnetic induction communication has been utilized for localization in water distribution systems. Kazeminasab and Banks (2021) synchronized a wireless sensor module localizer with the motion controller, enabling long-distance inspection capabilities. Kazeminasab and Banks (2022b) presented a localization method for in-pipe robots using a particle filter-based localizer synchronized with a multi-phase motion controller.

In comparison to these works, our approach aims to combine low-power exploration (using lower-power distance sensors and odometry) and vision-based topological localization for pipeline networks. We utilize low-power distance sensors for maintaining robot movement within the pipeline, while vision is employed at junctions for localization and construction of a topological map. Our focus is on lightweight underground pipeline robots, taking into consideration power and computation constraints. By integrating localization and motion control, we aim to achieve accurate and efficient smart motion for long-distance inspection in pipeline networks.

## 2 Materials and methods

The algorithm for automatic control is provided by Nguyen et al. (2022), and a detailed method overview can be found there. We present a brief discussion in Section 2.1. The junction recognition process uses normalized cross-correlation (NCC) image matching (Section 2.2). By comparing the current junction with all the junctions data collected in the database, the robot’s task is to determine whether the junction has been visited before, and if so, localize (Section 2.3). Otherwise, this new junction is added to the image database and the topological map is updated (Section 2.4).

### 2.1 Robot control for navigation and exploration

The miniature robot is equipped with three range sensors, an inertia measurement unit (IMU), two wheel-leg encoders, and a camera (800 × 800 Pixels) for sensing. The robot starts at the entrance of the pipe network and exhaustively explores all the sections before returning to the starting point. While exploring the pipe network, the robot encounters all junctions, dead-ends, and obstacles at least once, using its sensors to autonomously navigate and maneuver to deal with these conditions based on its estimated states. In our work and Nguyen et al. (2022), we do not use the camera for autonomous control of the robot but only fuse the data of the other three types of sensors to estimate the robot’s state. Eleven robot-in-pipe states capture different positions at T-junction, at left/right branches, at left/right corners, at straight pipe centerline, inclined in straight pipe, at a dead-end, at a cross junction, at open-space, and finally, to detect when the robot is approaching a collision. Three time-of-flight range sensors are installed at the front, front-left, and front-right of the robot to measure distances from the robot to the surrounding environment. These distances, combined with IMU data and their historical data, provide sufficient information for the algorithm to calculate the current robot’s local state (Nguyen et al., 2022).

Once the robot confirms its estimated local state, it makes a high-level decision to turn right or left at an angle, go straight, or turn around. By default, the robot decides to always take the right most unexplored branch at any junction to guarantee an exhaustive exploration of all sections of the pipe network. Specifically, the robot turns right at a cross junction, T-junction, right corner, and right branch. It goes straight at a left branch and in a straight pipe. It turns left at a left corner and turns around at a dead-end or upon encountering a significant obstacle (that blocks passage through the pipe cross-section). Depending on the direction of an impending collision, the robot turns left or right to move away from the pipe walls or obstacles. A detailed table of actions taken regarding the robot state was explained in Nguyen et al. (2022). After making a high-level decision, a low-level motor controller is called to fine-tune the robot’s direction and velocity in the pipes. Encoder values and their historical data are used to calculate the maneuver’s speed and estimated turning angles.

As described above, the robot uses three distance sensors mounted on the front to recognize a junction ahead. At this point, the robot faces the center of the junction ahead. In our work, we augment the control algorithm in Nguyen et al. (2022) to command the robot to move forward from the entrance to a junction by a set distance (equal to the estimated radius of the junction). Once at the approximate center of a junction, sampling, localization, and mapping take place, as described in the following sections. Once complete, the robot returns to the entrance of the junction, and the original control algorithm resumes.

We noted that the IMU, odometry and laser data are not used for localization but only for navigation and low-level control. Our localization method as described in this paper relies on vision information only.

### 2.2 Identification of known/unknown junctions

**FIGURE 1:**
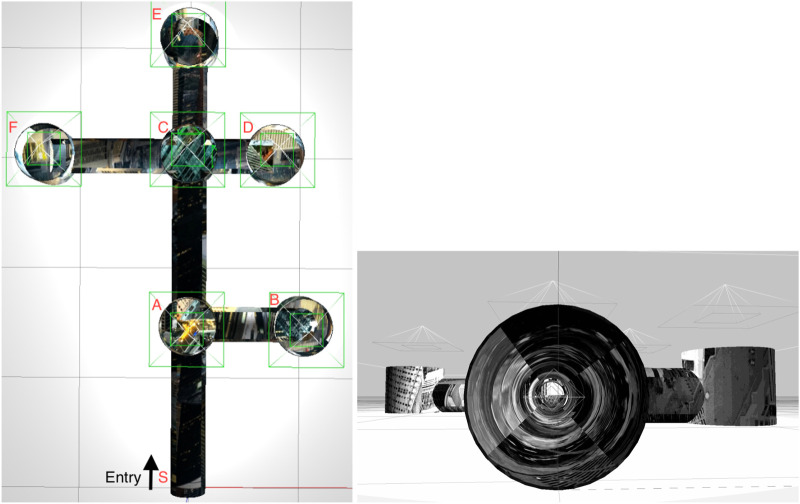
The simulated pipe network in the Gazebo simulator (Koenig and Howard, 2004). Pipe diameter 200 mm. Left: External view with the six junctions and entry point manually labeled. Right: inside view of a pipe with added texture.

The robot visits different junctions as it explores the pipe network (Figure 1). During this exploration, the robot may visit the same junction multiple times. We call such previously visited junctions *known junctions*. We call junctions that are encountered by the robot for the first time *unknown junctions*. This section describes how the robot can identify whether it is currently in a known or unknown junction. During autonomous exploration and topological mapping, the database consists only of known junctions. As more junctions are encountered, the database and map are updated.

**FIGURE 2:**
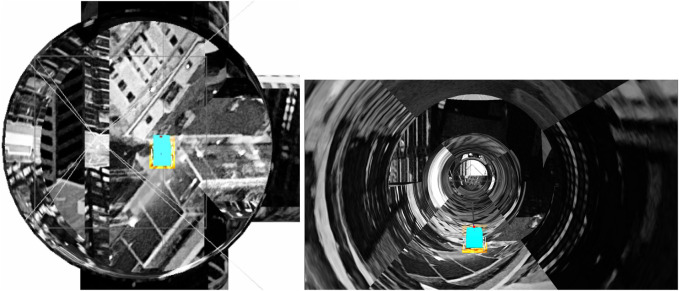
Robot (appearing as a light blue rectangle in a top-down and rear view) at the approximate center of junction A.

**FIGURE 3:**
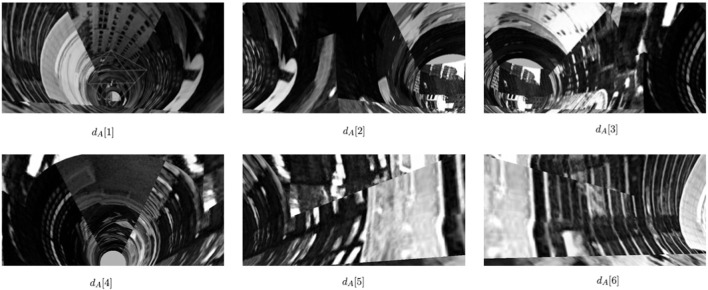
Collected image set from junction A (example of *d*_*A*_).

When the robot is approximately at the center of a junction (Figure 2), it collects images all around the junction (See example in Figure 3). The 360° view of the current junction is obtained by rotating the camera that is mounted above the robot (Figure 5) in discrete steps. Given the diameter of the simulated pipe, each image covers an angular width of about 90°. We want a significant overlap between images and found that a small set of 6 images (corresponding to 60-degree rotations between images) is sufficient for robust localization (see Section 3.2 in the Results). As the robot explores the environment, it collects images from each junction, and the stored sets of images are given by:

$$D={\left\{d_{i}\right\}}_{i=1}^{\text{NumJunctions}}$$

where *D* is the database, and NumJunctions is the number of known junctions at that point in the exploration.

To identify a junction, the robot compares the image set collected at the current junction, *d*_*c*_, with the image sets of all known junctions. Given two image sets *d*_*c*_ and *d*_*i*_, we perform image matching to define the similarity *S*(*d*_*c*_, *d*_*i*_) between the two junctions (see Algorithm 2 below). We then compute the maximum similarity score, *S*_max_, across all possible known junctions, to obtain the best candidate for a known junction, here labeled with index *j*, using:

$$\left(S_{\max},j\right)=\left(\max\left(S\left(d_{c},d_{i}\right)\right),\arg\underset{S}{\max}\right)$$

Finally, we determine whether the current junction is a known junction or an unknown junction by thresholding this maximum similarity score *S*_max_, shown in Algorithm 1. If the similarity is higher than the threshold, we identify the current location as a known junction *j* in the database. Otherwise, we perform active vision *A*(*d*_*c*_, *d*_*j*_) to better align the image sets (see Section 2.3; Algorithm 3) and repeat the image-set comparison with junction *j* in the database. If the similarity is still lower than the threshold, we define this junction as an unknown junction and add the current image set *d*_*c*_ to the database as a new junction. We also add this junction to the topological map, as described in Section 2.4.

Algorithm 1 Algorithm for identification of known/unknown junctions.

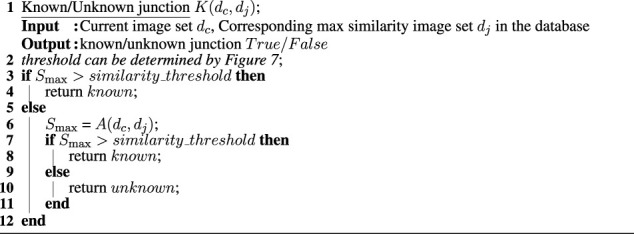

Normalized cross-correlation is used to calculate the maximum similarity, *S*(*d*_*c*_, *d*_*i*_), and corresponding offset between two different image sets. For greater realism, only grayscale information is used for image analysis. Image matching is performed for every possible junction pairing (one corresponding to the robot’s present location and the other for each ‘target’ junction in the database). There are six images in each image set, and hence six possible offsets between two image sets. For a given offset, the normalized cross-correlation is computed (in the Fourier domain) between all images in the current location with the candidate matching junction in the database. For each candidate junction, after finding the image offset with the maximum similarity, the horizontal pixel offset is set to the position of the maximum cross-correlation value. Looping through all possible candidate junctions, the pairwise similarity and the corresponding image and pixel shifts(offsets) are obtained. Finally, the highest similarity score is used to pin down a single candidate junction. The image-matching data for this junction pair is then used as a basis for the active vision step.

**FIGURE 4:**
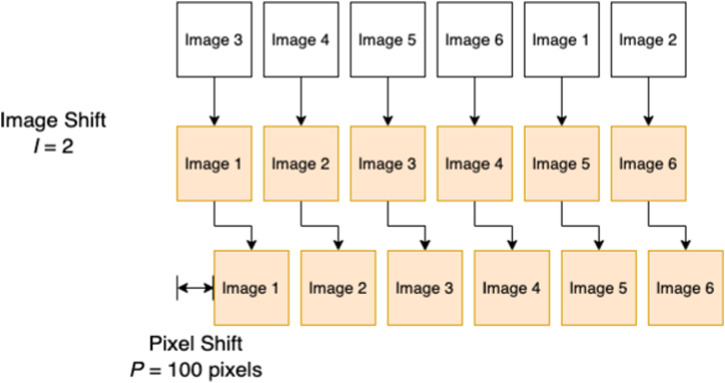
A illustration for the image shift *I* and pixel shift *P*. (white: currently sampled junction, orange: image set of a known junction from the stored dataset).

The normalized cross-correlation (NCC) is a commonly used region-based method in image feature matching. In the absence of a single 360° panoramic view of the entire junction, here we apply NCC to one image-pair at a time and increment the possible image shifts to allow for the robot entering the junction from different pipe sections, as illustrated in Figure 4. We match the similarity of gray pixel values in the whole image field in the two images (*I*_1_ and *I*_2_). For *I*_1_, we calculate the normalized cross-correlation coefficient between it and the *I*_2_, and the corresponding pixel shift that maximizes the match.

We exploit the similarity in the formulation of the cross-correlation to a convolution function, which can be implemented efficiently with a Fast Fourier Transform (FFT). To perform a cross-correlation, we perform a convolution between one image and a second conjugate image (see Algorithm 2). The implementation uses python3.6, opencv2, Numpy, and fftconvolve function from scipy.signal.

Algorithm 2 Algorithm for computing the similarity of two image sets and corresponding image shifts, pixel shifts.

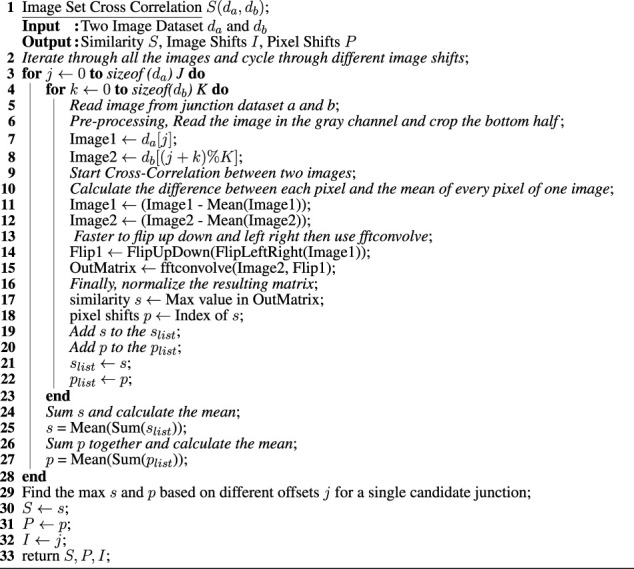

We note that a preprocessing step is taken before performing the cross-correlation. Because the lower part of the image contains the robot, which will affect the accuracy of the comparison results, we crop out the lower half of the image. Next, we subtract the mean pixel intensity.

### 2.3 Pipeline robot localization using active vision

As previously mentioned, a minimal change to this control algorithm was implemented to allow the robot to approach the approximate center of the junction. In this section, we describe a second addition to the control algorithm: the implementation of active vision.

Given the image shift I, pixel shift P, as well as the angle between successive images (2*π*/6 radians), camera width (2*π*/4.5), and pixel widths (800 pixels), the relative angle between the two image sets, is (I*2*π*/6 + P*cam_*width*/*pixel*_*width*). To execute the active vision, we rotate the camera according to this angle, and then move the robot a little distance forward, backward, left, and right, in turn from its position (roughly in the center of the junction). We continue cross-correlating with the same dataset in the database until we find a direction that increases the similarity. If active vision increases the similarity sufficiently to exceed to similarity threshold, the junction can be identified (it is a known junction). Otherwise, it is classified as an unknown (i.e., new) junction, as described in Section 2.2; (Algorithm 3).

Algorithm 3 Algorithm for using the active vision to find the maximum similarity of two image sets based on image shifts, pixel shifts.

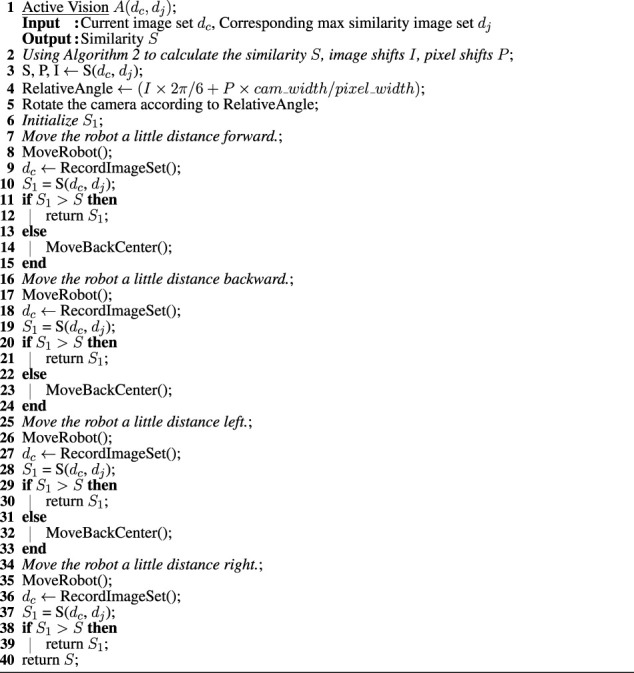

### 2.4 Topological mapping

Topological maps capture the relationships between elements of a map that are represented as nodes (or vertices), connected by edges (Garcia-Fidalgo and Ortiz, 2015). In our case, the map captures connectivity between pipe segments and the correct order of these edges in each junction. While distance information can be important for some purposes, the adjacency of pipe segments and junction information suffice for path planning in the network. The key to constructing topological maps is identifying appropriate spatial points of interest in the environment as vertices and extracting sufficient spatial semantic information from these locations. In addition to their compactness and elegance, topological maps may be more stable than spatial maps in the face of closed loops (in the absence of absolute position information). It is possible to consider a more spatially grounded topological map, in which edges (i.e., pipe sections) are assigned information (e.g., a distance estimate from dead reckoning, the time taken to move between two vertices, or the energy consumed). In our case, however, junctions contain sufficient semantic information about the scene.

Whether a junction is known or unknown dictates whether the topological map is up to date. Given an unknown junction, the robot firstly adds the image set *d*_*c*_ to the database *D*, creates a new vertex *V*_*n*_, and adds an edge *E* between it and the previous vertex the robot visited *V*_*last*_. When moving between two known junctions, the edge may or may not have been traversed previously. In the absence of an edge in the map, a new edge will be added between the previous junction to the current position. Topological maps typically encode vertices and edges in an adjacency graph. For navigation purposes, it is also useful to disambiguate the ordering of the edges for each junction. Here, we use the order in which edges are traversed to assign them integer labels (W). Whenever a new junction is added, if the edge used to reach the junction is new as well, it is assigned the next unused integer as its label. Thus, once the map is complete, each edge has a unique label (see Algorithm 4). Given the exploration algorithm (in our case, consistently taking the right-most branch), it is then possible to determine the relative orientation of the pipe sections.

Algorithm 4 Algorithm for processing a junction during topological map building.

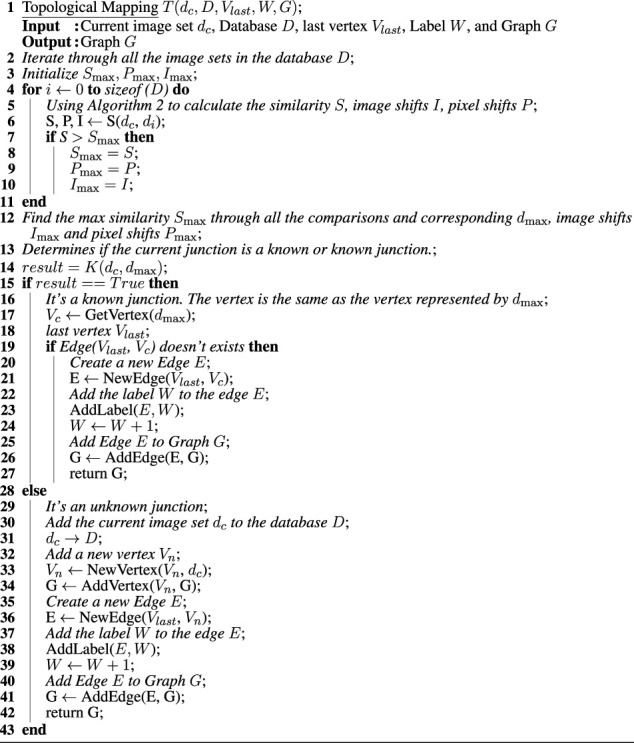

## 3 Results

In this section, we present our experiment with a simulated robot in a pipe environment. Our overall goal is to test the performance of our system by building a topological map doing autonomous control. However, before we present these topological map-building results in Section 3.6, we will present a set of simpler experiments that we have performed to test the localization performance of our robot. The following five experiments will be presented.

1. *Perfect Center*: Our first experiment tests if the robot can identify which junction it is in with respect to the junction in the database when the robot is initialized perfectly at the center of the junction. In this case, autonomous navigation is not used. We also use a robot initialized perfectly at the center of junctions to decide how many images to collect in each junction. The experiment suggests that six images are sufficient (see Section 3.2).
2. *Noise*: To validate that six images suffice even for the imperfect positioning of the robot in the center of the junction, we repeated the above experiments such that robot will be located at 3 cm off center (see Section 3.3).
3. *Active*: To test the robot’s ability to improve its localization through active vision, in this set of experiments, the robot is initialized at the off-center position and uses active vision (see Section 3.4).
4. *Entrance*: To more realistically simulate the process of the robot traveling autonomously in the pipeline, we place the robot on the edge of the junction in the pipeline, and perform a similar set of experiments. The robot moves forward a distance of the junction radius to the center before switching on its camera (see Section 3.5).
5. *Autonomous*: The robot is simulated under the fully autonomous exploration and control mode. A topological map is built (see Section 3.6).

### 3.1 Experimental setup

**FIGURE 5:**
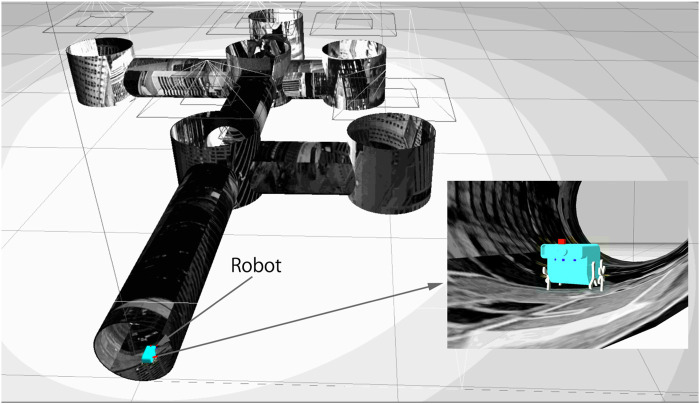
Robot simulated in the pipe network.

We model the mobile robot in a pipe network (Figure 1), and test our localization algorithm using ROS (Quigley et al., 2009) and the physics simulator Gazebo 9 (Koenig and Howard, 2004). The robot 3D model was designed in Solidworks (see Figure 5; Nguyen et al. (2022) for details of the design) and imported into Gazebo. The imported model in Gazebo is shown in Figure 5. The robot has six wheel-legs, with wheel diameter 28 mm. Three left wheel-legs are connected and are actuated by a DC motor. Similarly, three right wheel-legs are connected and controlled by the second DC motor. The two motors are independent and controlled by two PID (proportional integral differential) controllers. Each motor controller is implemented in Gazebo-ROS using the ros_control package (allowing complex joint control algorithm to be applied to the DC motor instead of standard differential drive).

Figure 5 shows the simulated pipe structure in Gazebo and the simulated pipe network with the robot. The grayscale texture was imposed on the simulation environment to ensure that junctions look distinct. This is an underlying assumption of our work–i.e. that there is a visual difference between geometrically identical junctions. In a pipe network of any age, this is likely to be true but needs to be experimentally verified in future work. The robot is controlled by sending commands to its two ros_control motor controllers. The simulation provides an idealized scenario for the robot model, in which we can develop and test the proposed algorithms for localization and mapping.

### 3.2 Initializing the robot at the center of the junction

We collected images for each junction, sampled with a robot positioned precisely at the center of each junction as a reference database. Figure 3 shows sample data collected from one junction.

**FIGURE 6:**
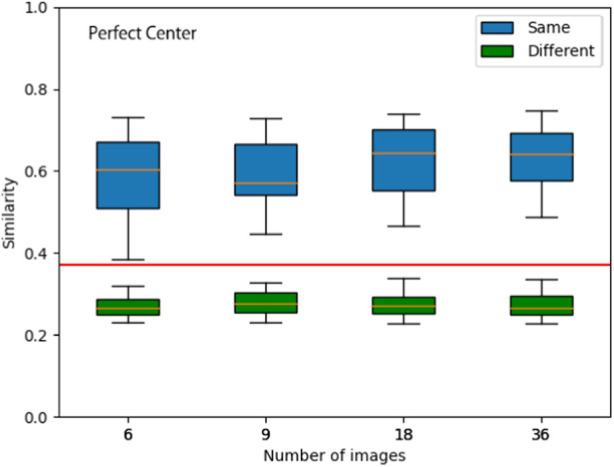
Assessing junction similarity for different sized image sets. Junction similarity results for 24 image sets (4 per junction at 90-degree intervals) under Perfect Center conditions. The similarity is compared for different numbers of images per image set. Boxplot (showing the Upper whisker, Upper quartile, Median, Lower quartile, and Lower whisker from the top to the bottom for the known junction (blue) and unknown junctions (green).) Applying a threshold of 0.36 (red horizontal line) would allow for accurate recognition of known junctions and distinguishing unknown junctions.

To determine a robust number of images that would suffice for localization, we performed a preliminary experiment, in which we placed the robot in the center of each of the six junctions in our network. As each image has a view of about 90°, more than four images would be required to ensure some overlap between adjacent images. We compared image sets with 6, 9, 18, and 36 images. For each case, four image sets were collected from each junction, facing four different directions (at 90-degree intervals) resulting in 24 image sets. We used the same robot to collect all the data sets required for the experiment at the same position in the pipeline. Defining the image sets from different robot orientations of the same junction as “Same” and image sets from different junctions as “Different.” we calculated the similarity scores to the Same and Different junctions for image sets with 6, 9, 18, and 36 images. We present the results in Figure 6. While increasing the number of images yields higher accuracy, even with only six images, junctions can be recognized and the rotational error is small (≲ 3°). The result demonstrates the robustness of the algorithm to the number of images used, with six images being sufficient to reliably identify junctions under perfect center conditions.

**FIGURE 7:**
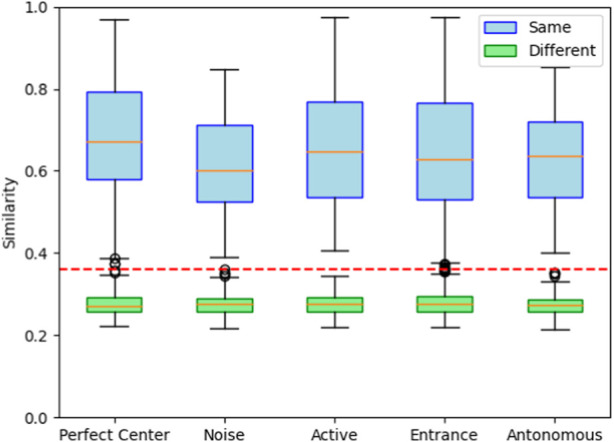
Image matching between junctions in the simulated pipe network (orange line: mean values, lower quartile: median of the lower half of the dataset, upper quartile: median of the upper half of the dataset, red line: threshold 0.36). Similarity scores for “Same” are computed as $S\left(d_{i^{1}},d_{i^{2}}\right)$ , where $d_{i^{1}}$ and $d_{i^{2}}$ are different imagesets captured from the same junction *i*. Similarity scores for “Different” are computed as *S*(*d*_*i*_, *d*_*j*_), where *d*_*i*_ and *d*_*j*_ are different imagesets captured from the different junctions, *i* and *j*. Same and different junctions can be robustly separate by the threshold.

To verify whether we can distinguish between known and unknown junctions, we collected sets of images for six junctions within the pipeline under different experimental conditions. For each experimental condition, we collected 5 imagesets at each junction giving us a total of 30 imagesets. We then compared the collected imagesets pairwise. Based on whether these sets of images originated from the same junctions or different junctions, we computed similarity results for junctions labeled as “known” and “unknown”. Notably, each known category comprises 60 data points(imageset pairs coming from same junction) in its box plot, while the unknown category encompasses 375 data points(imageset pairs coming from different junctions). These results are presented in Tables 1, 2; Figure 7.

**TABLE 1:** Summary of similarity results across different image sets under different conditions. Note the low similarity obtained for unknown junctions, across all conditions.

Similarity ± std	Perfect center	Noise	Active	Entrance	Autonomous
Known junction	0.700 ± 0.157	0.614 ± 0.112	0.675 ± 0.166	0.643 ± 0.150	0.631 ± 0.109
Unknown junction	0.276 ± 0.028	0.276 ± 0.027	0.276 ± 0.024	0.280 ± 0.031	0.273 ± 0.025

**TABLE 2:** Summary of rotation error across different image sets under different conditions.

Rotation error (radians)	Perfect center	Noise	Active	Entrance	Autonomous
Known junction	0.054	0.044	0.051	0.047	0.101
Unknown junction	0.377	0.473	0.610	0.717	0.856

### 3.3 Initializing the robot in the off-center junction position

To validate that six images suffice even for the imperfect positioning of the robot in the center of the junction, we repeated the above experiment for eleven image sets collected from robots that were located 3 cm off the center from a random direction (‘Noise’) (Tables 1, 2; Figure 7). Henceforth, all image sets consist of six images per junction. Next, we tested the robustness of our junction identification algorithm. To verify that the identification of ‘known’ junctions was reliable, we also checked the relative angular offset (obtained from the image and pixel shifts) and confirmed that these agreed with the different robot orientations.

### 3.4 Using active vision

Our next experiment tests the robot’s ability to improve its localization through active vision. While our noisy experiment still showed good results, we expect that more realistic pipe conditions may result in greater slippage or possible errors in estimating the radius of a junction. To address such conditions, here, we move the robot’s position to bring it closer to the position at the time of image set acquisition in the database (corresponding to previous visits to the known junction). Active vision dramatically improves the robot’s position recognition accuracy over the ‘noise’ experiment above.

### 3.5 Initializing the robot at the entrance of a junction

After setting up a simulation environment, we applied the same control methods described in Nguyen et al. (2022) to the simulated mobile robot. We updated the algorithm so the robot could move to the center of the junction and execute image collection for localization and mapping. The robot was able to explore the simulated pipe network exhaustively in the simulation environment, but also experienced failure in some maneuvers due to sensitivity to positioning, when resuming navigation of the network after visiting a junction. Here, we focus on results from successful runs.

To more realistically simulate the process of the robot traveling autonomously in the pipeline, we place the robot on the edge of the junction in the pipeline (‘Entrance’). The robot is oriented toward the center of the junction where its distance sensors detect a junction ahead while advancing in the pipeline. From this ‘entrance’ point, the robot moves forward at a fixed distance, set to be the radius of the junction. As in previous experiments, we collect image data at the position where the robot finally stops, which can better simulate the heading error in the real world. We collected eleven image datasets of robots after having moved autonomously from the edge to the center of the junction. A comparison of these eleven image sets’ data in the database is shown in Tables 1, 2; Figure 7.

From Table 1 results and using a threshold of 0.36 in the similarity, the robot can recognize the same junction, even off-center. In the third step of the active vision experiment, the robot achieves slightly improved mean similarities but is subject to larger variability. The robots from the junction entrance were all identified quite well and were considerably more accurate than the second and third sets of experiments.

### 3.6 Autonomously generating an ordered topological map

**FIGURE 8:**
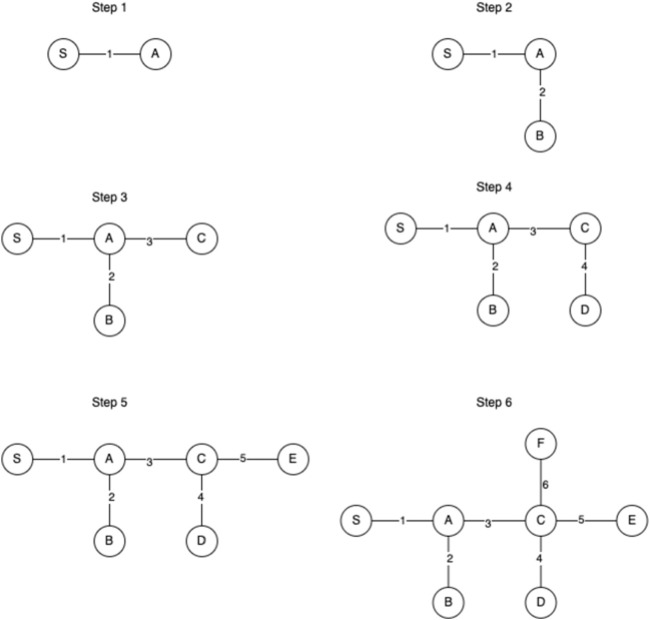
Dynamic topological map, generated during autonomous exploration of a simulated pipe network (shown in Figure 1). The six steps correspond to the six updates, each time a new (unknown) junction is visited. The edge labels represent the order in which the junctions were added to the map, and their relative orientations were determined with the knowledge of the rule-based exploration algorithm, yielding an ordering consistent with the pipe network. For visualisation, 90° angles were always assumed between adjacent edges since this is usually the case in real life situations.

We now test our method under fully autonomous conditions. For this experiment, the junction database is initially empty and is updated every time the robot traverses a new and different junction, from those previously visited. We conducted 7 experiments each starting at different position in the pipe network shown in Figure 1. The starting positions were: S,A,B,C,D,E,F. The robot was able to generate the correct topological map successfully from all of these starting positions. In Figure 8, we shown one example. The robot starts at entrance location S and exhaustively explores the network until it returns to the entrance. At every junction, based on the image database of previously visited junctions and the robot’s ability to recognize the current junction, the robot’s topological map is updated in real-time. A successful map is shown based on the above perfect junction sampling.

We define the topological map as an undirected graph in which each node represents a junction that was visited and imaged by the robot. Each time the robot identifies its location with a junction in the database, it successfully proceeds without updating the map. Conversely, when the robot arrives at a new, unfamiliar node, the current junction and its set of images are successfully added to the database, a new node is added to the graph, and an appropriately labeled edge connects the previously visited node to the current node (Figure 8).

## 4 Conclusion

Our work employs a multimodal sensing strategy to combine a miniature autonomous robot’s exploration, localization, and mapping in a simulated pipe network. While the results presented here are based on simulation experiments, we note that the robot is based on the SolidWorks model used to build a physical miniature model that successfully explored a similar pipe network. A key challenge in this work arises from the miniature robot’s limited mobility and limited power. Hence we sought an efficient (power-saving) strategy that requires relatively little computation and storage. We rely on our finding in (Nguyen et al., 2022) that the range sensor data in the physical robot is accurate and can make good estimates of the surrounding environment when the robot executes navigation and obstacle avoidance. We proposed that vision sensors are rich in data and we demonstrate that using fast normalized cross-correlation methods suffice in our experiments to identify environmental landmarks. We adapted traditional image-matching techniques to pipeline geometries and rotational movement of the camera. We further propose that active vision may be useful to compensate for the limitations of poor positioning of robots during realistic autonomous movement in pipeline environments. It is important to highlight that although a specific robot model was used in our simulated experiments, the vision-based localization technique we propose is designed to be compatible with various robotic systems, rather than being limited to a particular robot. As long as a robot is capable of exploring the pipe network and equipped with a camera, our method can be employed for topological mapping of the network.

Our work primarily focuses on developing image-matching algorithms for active vision. However, it is important to address the main limitation encountered with the physical robot, which relates to the location and motorization of the mounted camera. In future work, we plan to conduct tests and evaluations in a physical setting using either the same robotic platform or an alternative one that allows for panoramic imaging, enabling us to assess the performance of both the vision-based algorithm and the control mechanism. It should be noted that the sensors used in our work were studied solely in the simulation environment, and therefore, it is challenging to reliably estimate the accuracy of the robot in an actual physical setting. Furthermore, conducting experiments with physical robots in realistic pipe networks introduces additional complexities due to sensor limitations, robot stability, and control issues. It is crucial to acknowledge the significant challenges associated with system integration and electronic isolation in real-world pipe networks, which are often wet, dirty, and cluttered. To thoroughly evaluate the performance, reliability, and durability of the in-pipe robot, extensive testing and validation in real-world pipeline environments are essential. Another noteworthy challenge in real-world deployments is failure recovery. For instance, if the robot becomes stuck inside the pipe network, our current method does not provide a solution for rescuing it. Additionally, real-pipe networks often pose the challenge of dark lighting conditions. Consequently, in a practical deployment, the robot may require onboard lighting to support the camera’s visibility. Under such settings, the usefulness and feasibility of active vision can be more extensively tested. The experiments conducted in our work have primarily focused on a specific pipe texture. Conducting experiments in a physical pipe environment will enable us to test our proposed method with real pipe textures and verify our hypothesis that the distinct textures at junctions are sufficiently different to enable the differentiation of various junctions. In summary, while our work emphasizes image-matching algorithms for active vision, it is crucial to recognize and address the challenges posed by the physical robot’s limitations, system integration in real-world pipe networks, failure recovery, lighting conditions, and the need to validate the method with different pipe textures in physical pipe environments.

## Data Availability

Original datasets are available in a publicly accessible repository: The original contributions presented in the study are publicly available. This data can be found here: (https://github.com/SheldonLeeLXY/Simulated_Data).
